# Practical Algorithm Evaluating Preoperative Risk Factors for Posterior Capsule Rupture During Phacoemulsification

**DOI:** 10.7759/cureus.78907

**Published:** 2025-02-12

**Authors:** Eirini Oustoglou, Asimina Mataftsi, Lamprini Banou, Maria Dermenoudi, Nikolaos G Ziakas, Ioannis Tsinopoulos

**Affiliations:** 1 2nd Department of Ophthalmology, School of Medicine, Faculty of Health Sciences, Aristotle University of Thessaloniki, Thessaloniki, GRC; 2 Department of Ophthalmology, Health Center of Neapolis, Thessaloniki, GRC

**Keywords:** cataract surgery, complications, phacoemulsification, posterior capsule rupture, risk factors

## Abstract

Purpose: The purpose of the study is to create a practical and efficient tool for the preoperative assessment of cataract surgery based on statistical data.

Methods: A two-phase study was conducted in a tertiary teaching ophthalmology department, including a retrospective cohort for 2014-2015 and a prospective cohort for 2017-2018. In the first phase of the study, all preoperative files of cataract patients (excluding trauma, uveitis related and pediatric cataracts) were gathered and analysed for 2014-2015. An algorithm was created based on their preoperative assessment and then tested in a prospective cohort for 2017-2018, following the same inclusion criteria.

Results: The selection of predictors among the 1792 patients in the retrospective cohort was based on univariate and multivariate logistic regression analysis. The model with the lowest Akaike Information Criterion was formulated including three factors regardless of their p-value (age, sex, laterality) and the statistically significant factors, mature cataract, pseudoexfoliation, phacodonesis, diabetes, glaucoma, monocularity and resident surgeon at different rates of influence. The algorithm was tested in the prospective cohort (2017-2018) in 2057 cataract patients. The overall misclassification error rate was 5.9%, and the area below the ROC curve was 0.62 (CI 0.57-0.67).

Conclusions: The model created can assess patients and preoperatively evaluate their perioperative risk of complications while planning surgery with greater safety. Every population under study has unique characteristics, and safer assumptions can be made when particularities have been identified and taken into account. External validation would provide more information on its applicability in other teaching ophthalmology departments.

## Introduction

One billion people suffer from vision impairment that could have been prevented or needs to be addressed. The leading cause of vision impairment and blindness continues to be the cataract, with an estimated 100 million affected people. The World Health Assembly sets new goals to increase adequate coverage of cataract surgery to prevent that [1]. A similar improvement in the quality and safety of cataract procedures is anticipated, even though modern cataract surgery is already regarded as relatively safe. Cataract surgery remains the most common surgical operation in the European Union, with 4.3 million procedures in 2018 [2]. Maintaining a high-efficiency level in cataract removal and increasing patient safety addresses the ideal cost-effectiveness balance [3].

The preoperative assessment is essential to identify the risks and prepare every surgeon and patient for the surgery. Various preoperative stratification systems are used to prevent complications such as posterior capsule rupture with or without vitreous loss [4-6]. Surgical experience (volume) is considered crucial for challenging cases. Stratifying experiences and risk factors can help surgeons mature surgically through the direct connection of preoperative assessment with the intraoperative difficulty and the final result [7-9]. Combining these factors in a busy clinical practice sets the essential elements of safety, efficiency and practicality in the foreground. In this way, the incidence of cataract complications seems to decrease over time [8].

The primary aim of this study is to create a practical and efficient tool for the preoperative assessment of the risk of posterior capsular rupture during cataract surgery based on statistical data gathered from a tertiary, teaching ophthalmology department.

## Materials and methods

The study was conducted in two phases. The first phase was a retrospective cohort for 2014-2015, including anonymised data of all eyes undergoing cataract surgery through phacoemulsification (n=1792). Combined procedures were excluded along with pediatric, trauma and uveitis-related cataracts. The range of complications included posterior capsule rupture with or without vitreous loss. In the second phase, an algorithm was created from the statistical analysis, and the algorithm was tested in a prospective cohort from 2017 to 2018 (n=2058). The study was conducted in a single tertiary setting (2nd Department of Ophthalmology, School of Medicine, Faculty of Health Sciences, Aristotle University of Thessaloniki, Greece). The study protocol was approved by the Institutional Review Board of Papageorgiou General Hospital, the Ethics Committee of Aristotle University of Thessaloniki (ΑΠ. 3.394/02-05-2018), the 3rd Health District of Macedonia, Directorate of Human Resources Development of Health Services and Social Solidarity Units, Department of Continuing Education and International Cooperation (Δ3β/28889/02-08-2018) and adhered to the Declaration of Helsinki tenets. 

The preoperative risk factors studied were age >88years, axial length >26mm and <21mm, corneal scarring, posterior polar cataract, previous vitrectomy, anterior chamber depth <2.5mm, dilated pupil diameter <3mm, poor compliance, deep sulcus, α1 blockers intake, mature (brunescent/white/intumescent) cataract, phacodonesis, pseudoexfoliation, monocularity, glaucoma/ocular hypertension under treatment and diabetes mellitus. The majority of the factors above were based on a model created by Muhtaseb et al. [4] and four more were added based on a literature review of cataract complications.

In Phase One, the preoperative examination was conducted by a single experienced clinician (EO). All risk factors were documented on a specific form along with the patient’s medical history and measurements from the IOL Master 500 (Carl Zeiss Meditec AG, Jena, Germany). Intraoperative complications were recorded on the electronic operation room (OR) protocol shortly after the end of each surgery. Two independent investigators (E.O., M.D.) thoroughly reviewed all patient files and electronic OR notes. They recorded the presence of any preoperative risk factors, patient demographics and data regarding intraoperative complications and phacoemulsification metrics. 

In Phase Two, the same clinician conducted the preoperative assessment and followed the post-operating OR notes. The algorithm created from Phase One was easily managed from an Excel sheet during preoperative assessment. All the anonymised data were collected for statistical analysis.

Residents operate on eyes lacking risk factors at the beginning of their training and move on to patients with a history of diabetes and glaucoma. Along with the assessed factors, surgeries performed by residents were documented and included in the statistical analysis as risk factors in both phases. 

Statistical analysis

Descriptive statistics were calculated for demographic characteristics, predictors, and outcomes. Mean and standard deviation were used for continuous variables, and frequencies and percentages were used for categorical variables. 

The model was developed using multivariable logistic regression with a binary (complication/no complication) outcome. All candidate predictors (risk factors) were binary variables (presence/absence): vitrectomy, cornea scarring, small pupil, shallow anterior chamber, age over 88 years, ametropia, polar cataract, dense/total/white or brunescent cataract, pseudoexfoliation, phacodonesis, diabetes, glaucoma, deep orbit, α1 blockers intake, poor cooperation, and monocularity. For predictor selection, univariate logistic regression analysis was used. Predictors with a p-value less than 0.20 were included in the final model. Variables "age", "sex", and "laterality (left/right)" were considered confounding factors and, thus, they were included in the final model regardless of their p-value from the univariate analysis. The predictor "age over 88 years" was not included in the analysis as it was judged preferable to include the age of patients as a quantitative variable in the model. The predictors considered to be clinically significant were included in the final model regardless of their statistical significance. The final model was created based on the Akaike Information Criterion (AIC) value. The model with the lowest AIC value is the better one.

The overall model performance was evaluated based on two main characteristics: discrimination and calibration. Discrimination is the ability of a model to differentiate between patients who have experienced the outcome from those who have not. The measure used to assess the discrimination is the area under the receiver-operating-characteristic curve (AUC). An AUC value of 0.5 indicates that the model cannot discriminate between those two groups, while a value of 1 indicates an excellent model. Calibration is an assessment of the agreement between the predicted probabilities of the model and the observed probabilities of the data. The model calibration was evaluated using graphical methods along with the slope of the calibration curve.

The internal validation of the model was assessed using the Bootstrap method. The Bootstrap method is a sampling process in which a sample of n observations is selected from the original data set repeatedly, and the model is evaluated on each copy. The Bootstrap samples are the same size as the original sample. To validate the model, 500 Bootstrap samples with replacements were used.

## Results

The first phase of this study included 1792 eyes. The mean age was 72.8 (SD 9.3) years, 52.5% were women, and 49.1% were operated in the left eye. The overall complication rate for 2014-2015 was 6.7% (Table 1).

**Table 1:** Studied Preoperative Parameters ^a^Axial Length, ^b^Pseudoexfoliation, ^c^Ocular Hypertension

Variables	Total (%)
Mean Age (SD)	72.8 (9.3)
Sex, N (%)	
Male	851 (47.5)
Female	941 (52.5)
Eye, N (%)	
Left	880 (49.1)
Right	912 (50.9)
Complications, N (%)	
No	1672 (93.3)
Yes	120 (6.7)
Vitrectomy, N (%)	
No	1759 (98.2)
Yes	33 (1.8)
Corneal Scarring, N (%)	
No	1739 (97.0)
Yes	53 (3.0)
Small Pupil, N (%)	
No	1772 (98.9)
Yes	20 (1.1)
Anterior Chamber Depth (<2.5mm), N (%)	
No	1680 (93.8)
Yes	112 (6.3)
AL^a^>26mm and <21mm, N (%)	
No	1732 (96.7)
Yes	60 (3.3)
Polar Cataract, N (%)	
No	1756 (98.0)
Yes	36 (2.0)
Age > 88yo, N (%)	
No	1766 (98.5)
Yes	26 (1.5)
Mature Cataract (Brunescent/White/Intumescent), N (%)	
No	1582 (88.3)
Yes	210 (11.7)
PEX^b^, N (%)	
No	1565 (87.3)
Yes	227 (12.7)
Phacodonesis, N (%)	
No	1761 (98.3)
Yes	31 (1.7)
Diabetes Mellitus, N (%)	
No	1400 (78.1)
Yes	392 (21.9)
Glaucoma/OHT^c ^under Treatment, N (%)	
No	1601 (89.3)
Yes	191 (10.7)
Deep Sulcus, N (%)	
No	1704 (95.1)
Yes	88 (4.9)
α1 Blocker Intake, N (%)	
No	1609 (89.8)
Yes	183 (10.2)
Poor Compliance, N (%)	
No	1741 (97.2)
Yes	51 (2.8)
Monocularity N (%)	
No	1721 (96.0)
Yes	71 (4.0)
Resident, N (%)	
No	1573 (87.8)
Yes	219 (12.2)

Among all factors tested in the univariable logistic regression analysis, axial length >26mm and <21mm, mature cataract, pseudoexfoliation, phacodonesis, diabetes mellitus, glaucoma, being monocular and undergoing resident-performed surgery were those included in the multivariable logistic regression analysis, together with sex, age and laterality variables. A variety of multivariable models was created, and the one with the lowest AIC (AIC = 801.51) was considered the best. The final model was formulated as follows: 

Log Odds (Y=1|X) = -5.972 + 0.040 * age - 0.613 * sex - 0.206 * eye + 1.144 * mature cataract- 0.552 * pseudoexfoliation + 1.149 * phacodonesis + 0.441 * diabetes + 0.854 * glaucoma- 1.103 * monocularity + 1.802 * resident surgeon

Memo 

Binary variables are 1= Female, right eye, mature cataract, pseudoexfoliation, phacodonesis, diabetes, glaucoma, monocularity, and resident surgeon.

The continuous variable is age.

According to the model, the probability of complication is higher in patients with mature cataracts, phacodonesis, diabetes, and glaucoma and patients operated by residents (Table 2).

**Table 2:** Single Variable and Multivariable Analysis of the Preoperative Variables ^α^Odds Ratio, ^b^Confidence Interval, ^c^Axial Length, *p-value<0.05

Risk Factors	Univariable Regression Analysis			Multivariable Logistic Regression Analysis		
	OR^α^	95% CI^b^	p-value	OR^α^	95% CI^b^	p-value
Age (years)	1.04	1.02-1.07	0.002*	1.04	1.02-1.07	0.002*
Sex						
Male	1			1		
Female	0.63	0.43-0.91	0.015*	0.54	0.36-0.80	0.002*
Eye						
Left	1			1		
Right	0.81	0.55-1.17	0.25	0.81	0.55-1.20	0.3
Vitrectomy						
No	1					
Yes	1.4	0.42-4.67	0.58			
Corneal Scarring						
No	1					
Yes	0.54	0.13-2.24	0.39			
Small Pupil (<3mm)						
No	1					
Yes	1.56	0.36-6.79	0.56			
Anterior Chamber depth (<2.5mm)						
No	1					
Yes	0.92	0.42-2.03	0.85			
AL^c^>26mm and <21mm						
No	1					
Yes	0.23	0.03-1.67	0.15			
Posterior Polar Cataract						
No	1					
Yes	1.27	0.38-4.21	0.69			
Mature Cataract (Brunescent/White/Intumescent)						
No	1			1		
Yes	2.24	1.41-3.55	0.001	3.14	1.85-5.20	<0.001*
Pseudoexfoliation						
No	1			1		
Yes	0.61	0.31-1.18	0.14	0.58	0.27-1.12	0.13
Phacodonesis						
No	1			1		
Yes	3.47	1.39-78.62	0.007	3.16	1.06-8.16	0.025*
Diabetes Mellitus						
No	1			1		
Yes	1.45	0.96-2.19	0.08	1.55	1.00-2.38	0.046*
Glaucoma						
No	1			1		
Yes	1.53	0.90-2.58	0.11	2.35	1.30-4.08	0.003*
Deep Sulcus						
No	1					
Yes	1.22	0.55-2.58	0.11			
α1 Blocker Intake						
No	1					
Yes	1.28	0.73-2.23	0.39			
Poor Compliance						
No	1					
Yes	1.54	0.60-3.94	0.37			
Monocularity						
No	1			1		
Yes	0.39	0.09-1.63	0.19	0.33	0.05-1.16	0.14
Resident Surgeon						
No	1			1		
Yes	4.36	2.89-6.55	<0.001	6.06	3.82-9.59	<0.001*

**Figure 1:**
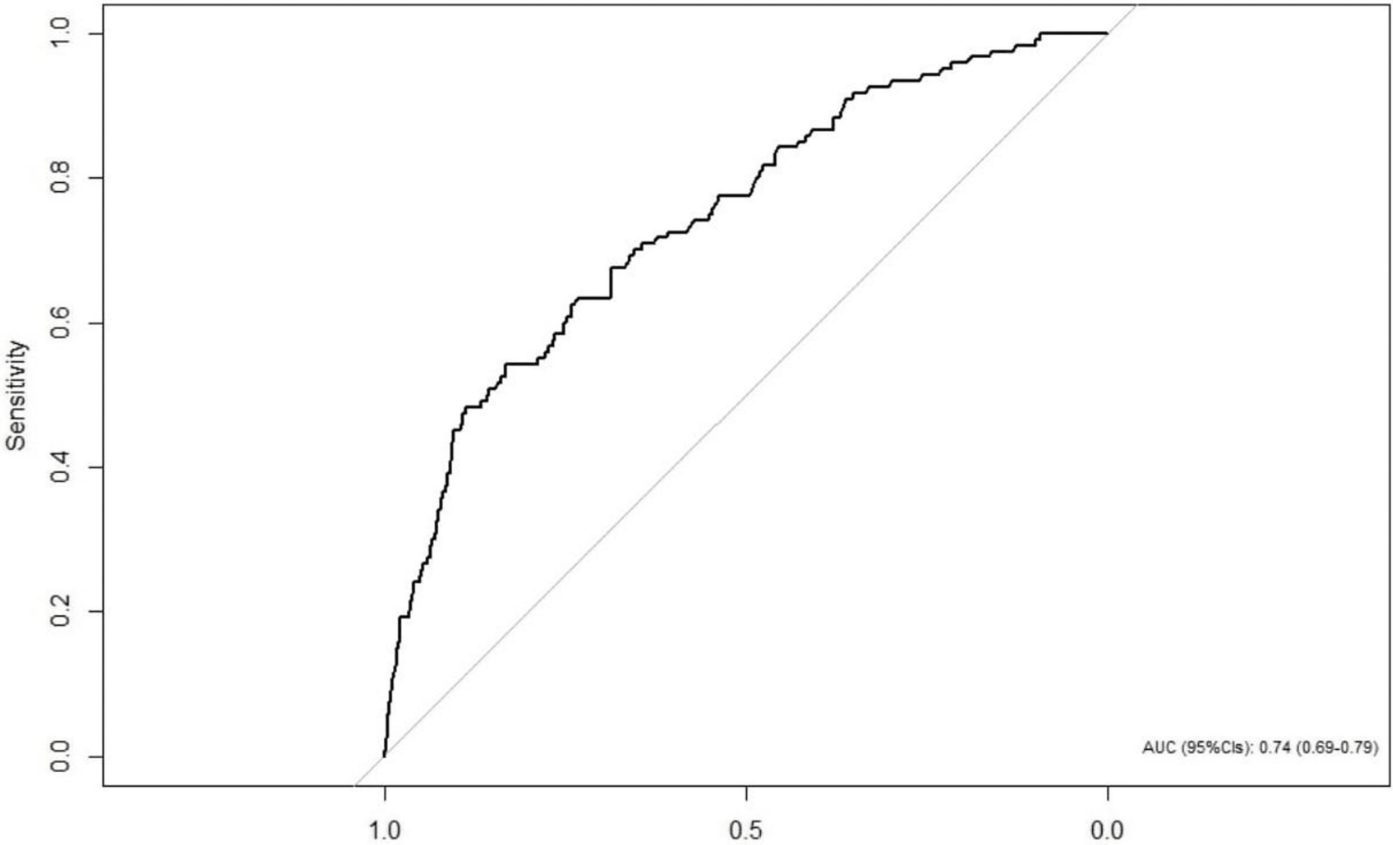
ROC Curve, Sensitivity and Specificity Area Under the Curve=0.74 (N=1792)

Figure 1 presents a ROC curve. The AUC was calculated at 0.74 with a confidence interval of 0.69 to 0.79.

The AUC was considered satisfying, and a similar estimate was obtained in the internal validation with the Bootstrap method with 500 replications (AUC = 0.76) (Table 3).

**Table 3:** Internal Validation (Ν=1792) ^α^Confidence Interval

	Final Algorithm	Bootstrap Method (500 repetitions)
Calibration Gradient	1	1
AUC (95%CI^α^)	0.74 (0.69-0.79)	0.76

**Figure 2:**
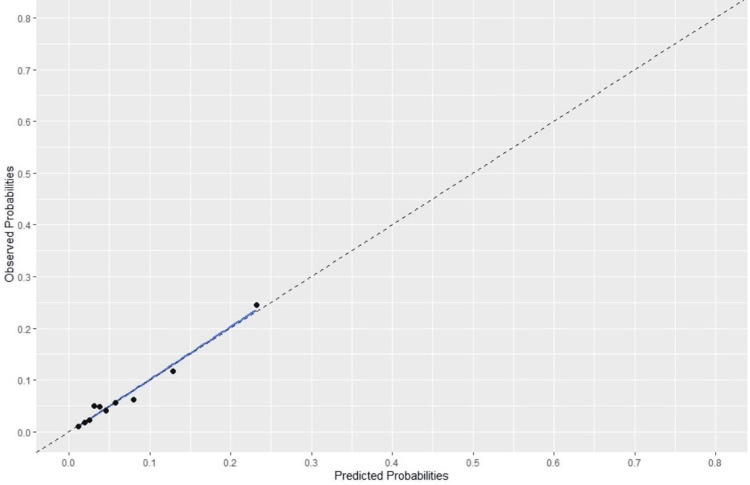
Predicted Probabilities against Observed Probabilities The pointing dots show the outcome observed (N=1792)

Figure 2 shows the adjustment graph of the outcomes observed against model predictions. The values ​​of the outcomes per decimals are given with dots.

After the Hosmer-Lemeshow calibration test, a p-value of 0.85 was obtained. Therefore, there is no indication of a difference between the observed outcomes and the outcomes estimated by the model. This p-value indicates a good calibration of the model. The calibration gradient is 1, which indicates a good calibration of the final model and after internal validation by applying the Bootstrap method (500 repetitions), the same value was obtained (Table 3).

The algorithm was then applied to clinical data collected prospectively in 2017-2018 concerning 2057 patients. The overall misclassification error rate for this model was 5.9%. In addition, the AUC is 0.62 with a 95% confidence interval (0.57-0.67), which is considered satisfactory. The comparison of demographic data between the two phases revealed a significant difference in the mean age of the study participants (72.8 - 73.5, p-value= 0.017). Although not significant, the complication rate decreased from 6.7% to 5.9% (p=0.331) in the prospective phase (Table 4). 

**Table 4:** Compared Demographics ^a^Independent samples t-test, ^b^Chi-square, *Statistically significant at 5% level

	Data From 2014 to 2015 Ν=1792	Data From 2017 to 2018 Ν=2057	p-value*
Age, mean (SD)	72.8 (9.3)	73.5 (8.8)	0.017 ^a^
Gender, Ν (%)			
Male	851 (47.5)	924 (44.9)	0.114 ^b^
Female	941 (52.5)	1133 (55.1)	
Eye, Ν (%)			
Left	880 (49.1)	1032 (50.2)	0.501 ^b^
Right	912 (50.9)	1024 (49.8)	
Complication, Ν (%)			
No	1672 (93.3)	1935 (94.1)	0.331 ^b^
Yes	120 (6.7)	122 (5.9)	

## Discussion

This study created a practical and efficient tool for the preoperative assessment of cataract patients, based on clinical data from a tertiary teaching ophthalmology department. Since every population under study has unique characteristics, safer assumptions can be made only when particularities have been identified and taken into account. The model we created was applied to patients envisaging cataract surgery from Northern Greece, a geographical area reflecting the service our department provides. The model can serve to assess patients and preoperatively evaluate their perioperative risk of complications, and it can therefore aid in planning surgery with greater safety.

The risk factors that appeared significant in the single-factor analysis were then taken into the multivariable logistic regression model. Except for the factors intentionally selected to appear in the final model (age, laterality and sex), only eight of the 16 achieved statistically significant levels in the multivariable logistic regression analysis. Ten of all factors (7/8 significant and the three intentionally selected) were chosen for use in the final model, based on the combination that served the lowest AIC [10]. The incidence of some risk factors within the sample remained low, contributing to their lack of statistical significance. Under no circumstances should risk factors such as α1 blockers intake, shallow anterior chamber depth, and high ametropia be taken lightly. Their inability to contribute more to the rate of complications probably has more to do with the familiarity of the surgeons rather than their low contribution to risk. Numerous recent studies emphasise the importance and probable dangers of α1 adrenergic blockers intake to men and women [11,12]. The shallow anterior chamber has long been described as associated with capsule complications and endothelial cell loss [13,14]. The extreme axial length of an eye, often coexisting with other ocular comorbidities, seems to be losing its primary high capsule complication rates, especially in the myopic end, in large series of patients [15].

Small pupils <3mm are a threshold rarely found in patients with common senile cataracts and therefore, in our sample, they represented only 1.1% of the patients. In these few eyes, mechanical stretch was applied to the iris margin (hooks, rings). This low threshold used in the preoperative assessment is based on the stratification system Muhtaseb et al. created and should not be considered as a limit beyond which a mechanical stretch device can be used [4]. Based on their experience, every surgeon should operate in the most comfortable possible environment to have fewer complications. The least comfortable pupil to be operated on without pupil expansion devices should probably have a 5mm limit [16,17]. 

Possible associations between resident-performed surgeries and the presence of diabetes and glaucoma were tested for their coexistence in the final model. The Z test found no association in the multivariable model, testing two percentages (p<0.001). The severity of the two conditions was neither documented nor stratified for each case; therefore, this works as a limitation and could yet be analyzed to determine the increased incidence of capsule complications in their presence.

This study’s strength is the two-phase design, aiming to confirm findings from the retrospective analysis in a prospective trial in the same setting. The model has the advantage of combining statistically significant risk factors, age as a continuous variable, and the option of a specific surgeon level. This model can be evaluated, adjusted if needed and implemented in any teaching hospital. It is relatively quick, practical, and easily included in a computerized system. Its goal is to document risk factors, assess the odds of possible complications, inform the patients and prepare the surgeon for the case to come.

The lack of a national registry reduces the possibility of creating a model through a larger scale of patients. At such a level, all the risk factors studied could be represented in the final model. The limited number of statistically significant risk factors is a considerable limitation to the application of the study. The model created regards a specific ophthalmic unit and reflects the population of a particular geographical area. As such, every region willing to use it should first test it to apply its demographic characteristics. 

## Conclusions

The algorithm created based on the internal validation seems to predict the outcome satisfactorily. Based on its overall misclassification error rate, this model is good enough to predict whether a person will have an intraoperative complication or not, as the lower the percentage, the better the model is able to predict the outcome. However, external validation would provide more information on its applicability in other teaching ophthalmology departments.
